# Relationship between IHC4 score and response to neo-adjuvant chemotherapy in estrogen receptor-positive breast cancer

**DOI:** 10.1007/s10549-017-4266-9

**Published:** 2017-04-26

**Authors:** A. Sheri, I. E. Smith, M. Hills, R. L. Jones, S. R. Johnston, M. Dowsett

**Affiliations:** 1 Royal Free Hospital, Pond Street, London, NW3 2QG UK; 2 Royal Marsden Hospital, Fulham Road, London, SW3 6JJ UK; 3 Institute of Cancer Research, Fulham Road, London, SW3 6JB UK

**Keywords:** IHC4, Ki67, Neo-adjuvant chemotherapy, Breast cancer

## Abstract

**Aims:**

To determine whether IHC4 score assessed on pre-treatment core biopsies (i) predicts response to neo-adjuvant chemotherapy in ER-positive (ER+) breast cancer; (ii) provides more predictive information than Ki67 alone.

**Methods:**

113 patients with ER+ primary breast cancer treated with neo-adjuvant chemotherapy at the Royal Marsden Hospital between 2002 and 2010 were included in the study. Pathologic assessment of the excision specimen was made for residual disease. IHC4 was determined on pre-treatment core biopsies, blinded to clinical outcome, by immunohistochemistry using quantitative scoring of ER (H-score), PgR (%) and Ki67 (%). Determination of HER2 status was made by immunohistochemistry and fluorescent in situ hybridization for 2+ cases. IHC4 and Ki67 scores were tested for their association with pathological complete response (pCR) rate and residual cancer burden (RCB) score.

**Results:**

18 (16%) of the 113 patients and 8 (9%) of the 88 HER2-ve cases achieved pCR. Ki67 and IHC4 score were both positively associated with achievement of pCR (*P* < 10^−7^ and *P* < 10^−9^, respectively) and RCB0+1 (*P* < 10^−5^ and *P* < 10^−9^, respectively) following neo-adjuvant chemotherapy in all patients. Rates of pCR+RCB1 were 45 and 66% in the highest quartiles of Ki67 and IHC4 scores, respectively. In ER+HER2-ve cases, pCR+RCB1 rates were 35% and in the highest quartile of both Ki67 and IHC4. There were no pCRs in the lower half of IHC4 or Ki67 scores.

**Conclusions:**

IHC4 was strongly predictive of pCR or near pCR in ER+ breast cancers following neo-adjuvant chemotherapy. Ki67 was an important component of this predictive ability, but was not as predictive as IHC4.

**Electronic supplementary material:**

The online version of this article (doi:10.1007/s10549-017-4266-9) contains supplementary material, which is available to authorized users.

## Introduction

The EBCTCG overview of clinical trials comparing adjuvant chemotherapy with neo-adjuvant chemotherapy in patients with primary breast cancer indicated that the benefit of chemotherapy is related to absolute risk. The potential benefit of chemotherapy in estrogen receptor (ER)-positive disease is therefore related to the residual risk on appropriate endocrine therapy [1]. Accurate determination of prognosis on adjuvant endocrine therapy and identification of patients at sufficiently low risk who might be spared adjuvant chemotherapy have been the priority area of recent research [2].

IHC4+C is a composite score incorporating immunohistochemical parameters ER, progesterone receptor (PgR), HER2 and Ki67 (IHC4) with a clinical treatment score (C) of tumour size, grade, nodal status and type of endocrine therapy developed on samples from the TransATAC cohort [3]. IHC4+C has been shown to provide similar amounts of prognostic information to that provided by the 21-gene recurrence score Oncotype DX, which has been approved by the National Institute for Health and Care Excellence (NICE) in the UK for guiding chemotherapy decisions in lymph node-negative ER breast cancer determined to be at intermediate risk of recurrence using standard clinical variables [3, 4].

A recent decision impact study reported that the use of IHC4 +C was associated with a reduced use of adjuvant chemotherapy in borderline cases where the benefit of chemotherapy was considered uncertain by clinicians using standard clinical variables [5]. Optimal clinical decision-making incorporates the use of both prognostic markers, which identify risk of recurrence together with predictive markers, which identify benefit from a particular therapy. Oncotype DX has been shown to predict the magnitude of chemotherapy benefit in tamoxifen-treated patients in the NSABP B20 trial although this study was limited by its small size, the overlap in the test set with the training test for Oncotype DX, and this being a secondary analysis [6].

The opportunities to assess the ability of prognostic scores to predict benefit from chemotherapy in the adjuvant setting are very limited because of the absence of good sample sets from randomized studies with a no chemotherapy arm.

The neo-adjuvant setting is an opportunity to obtain some analogous data for chemotherapy benefit. pCR following neo-adjuvant chemotherapy is established as an intermediate marker of long-term outcome, although ER-positive (+) breast cancers are far less likely to achieve a pCR [7–9]. In a small study of patients with locally advanced breast cancer, a higher 21-gene recurrence score predicted for pCR (*P* = 0.005). pCR was more likely with higher expression of proliferation-related genes and immune-related genes and with lower expression of ER-related genes [10]. However, no such data for IHC4 have been reported.

More recently, characterizing residual disease following neo-adjuvant chemotherapy beyond simple dichotomous classification of pCR or not has been described as the residual cancer burden (RCB) Symman’s et al. [11, 12]. This score has been shown to be prognostic and its performance is enhanced by its combination with post-treatment Ki67 to form the residual proliferative cancer burden (RPCB) [11, 12]. ER+ disease in particular has been associated with a favourable long-term outcome even in the presence of minimal or minimal proliferative disease following neo-adjuvant chemotherapy, i.e. low RCB or RPCB [12].

The development of these intermediate markers in the neo-adjuvant setting provides an opportunity to study the predictive role of pre-treatment biomarkers such as IHC4. Given that so far the IHC4 score has only been shown to be prognostic, the aims of this project were (i) to determine whether IHC4 score determined on pre-treatment core biopsies predicted response following neo-adjuvant chemotherapy in ER+ breast cancer; (ii) to determine whether IHC4 provided more predictive information than Ki67 alone, given that Ki67 has already been found to predict for pCR.

## Materials and methods

### Patient cohort

A total of 113 ER+ patients treated with neo-adjuvant chemotherapy for primary breast cancer at the Royal Marsden with pre- and post-treatment samples available were included in the study (Online Resource 1). Patients were identified retrospectively as a consecutive cohort from a prospectively maintained hospital research database between 2002 and 2010 [12]. Patients with stage IV disease were excluded, as were those with insufficient pathological material available for review.

### Clinical and pathological assessment

Clinical assessment of the primary tumour and lymph nodes was made according to WHO criteria using bi-dimensional caliper measurements of the primary tumour and axillary nodes. [13]. RCB assessment was undertaken on the post-treatment excision specimen as described previously to assess pathological response to neo-adjuvant chemotherapy [12]. This involved assessment of the bi-dimensional residual tumour bed, residual tumour cellularity, and extent of lymph node involvement including the number of lymph nodes involved and size of the largest lymph node metastases [11, 12]. Immunohistochemical staining was performed for ER, PgR and Ki67 on sections on 4-micron sections of formalin-fixed paraffin-embedded tissue from pre-treatment core biopsies as reported by Cuzick et al. to determine IHC4 [3].

### Statistical analysis

The primary objective of the study was to determine whether IHC4 determined on pre-treatment core biopsies was predictive of chemotherapy response. A secondary analysis was to determine whether IHC4 provided more prognostic information than Ki67 alone.

IHC4 was calculated according to the published algorithm by Cuzick et al. [3]. ER was quantified by the H-score and divided by 30 to arrive at a variable between 0 and 10 (ER_10_). PgR was quantified by the percentage of cells staining positive, and this was divided by ten to obtain a variable between 0 and 10 (PgR_10_). Ki67 was quantified as a percentage. As was described in the independent validation cohort, the Ki67 percentage was multiplied by 0.4 to scale for the difference between the image analysis technique used in the original development of IHC4 and manual scoring [14]. This was because Ki67 scores are on average 2.5 times higher with manual reading than using the image analysis method from which the algorithm was derived. HER2 was scored as positive if 3+ by IHC and equivocal 2+ samples underwent fluorescent in situ hybridization analysis and were considered positive if the ratio was two or more.

## Results

### Patient demographics and treatment

Baseline patient demographics are shown in Table 1. All 113 cases were evaluated for IHC4. Of these, 18 (16%) had achieved a pCR. Median age was 49. Almost all had T2 tumours or greater with just over half being clinically node positive at presentation. Fifty-six percent were grade 2, 37% grade 3 and 19% were HER2 positive. Treatment details are summarized in Table 1. Ninety-seven percent received a neo-adjuvant anthracycline and 66% received a neo-adjuvant taxane. Thirteen of the 22 HER2+ cases received neo-adjuvant trastuzumab. The majority underwent breast-conserving surgery. Median follow-up was 5.1 years and there were 26 relapses and 20 deaths.

**Table 1:** Baseline patient characteristics and treatment details (*N* = 113)

Characteristic	Total (*n*)	%
Age	Median 49	
Menopausal status		
Pre	65	58
Post	34	30
Unknown	14	12
T stage T0		
T1	1	1
T2	0	0
T3	63	56
T4	31	27
Unknown	14	12
N stage		
N0	49	44
N1	60	54
N2	1	1
N3	1	1
AJCC stage		
1a	0	0
1b	0	0
2a	38	34
2b	39	35
3a	19	17
3b	13	11
3c	2	2
PgR status		
Negative	27	24
Positive	73	65
Unknown	13	12
HER2 status		
Negative	89	79
Positive	22	19
Unknown	2	2
Grade		
I	5	4
II	63	56
III	42	37
Unknown	3	3
Histology		
IDC	101	89
ILC	10	9
Mixed	2	2
Other		
Surgery		
Breast conservation	63	56
Mastectomy	50	44
Unknown		
Neoadjuvant therapy		
Anthracycline	110	97
Taxane	75	66
Trastuzumab	13	12
Adjuvant taxane	11	1
Adjuvant endocrine therapy	111	98

### Response to neo-adjuvant chemotherapy

The relationship between pCR and RCB and long-term outcome in this ER+ cohort is shown in Online Resource 2. Outcome was significantly correlated with RCB class; those with RCB class 3 residual disease had a significantly poorer outcome following neo-adjuvant chemotherapy. This confirmed the expected relationship between RCB and long-term outcome in this cohort.

**Fig. 1:**
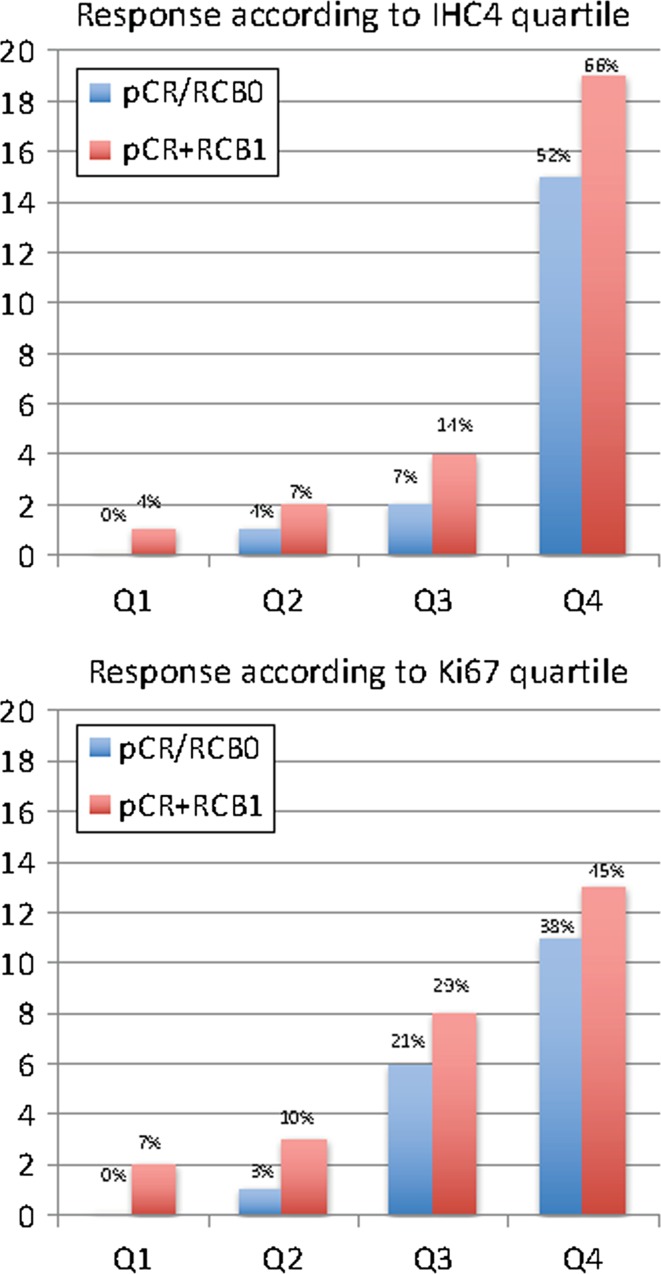
Pathological response according to IHC4 and Ki67 quartile for all cases (*N* = 113)

**Fig. 2:**
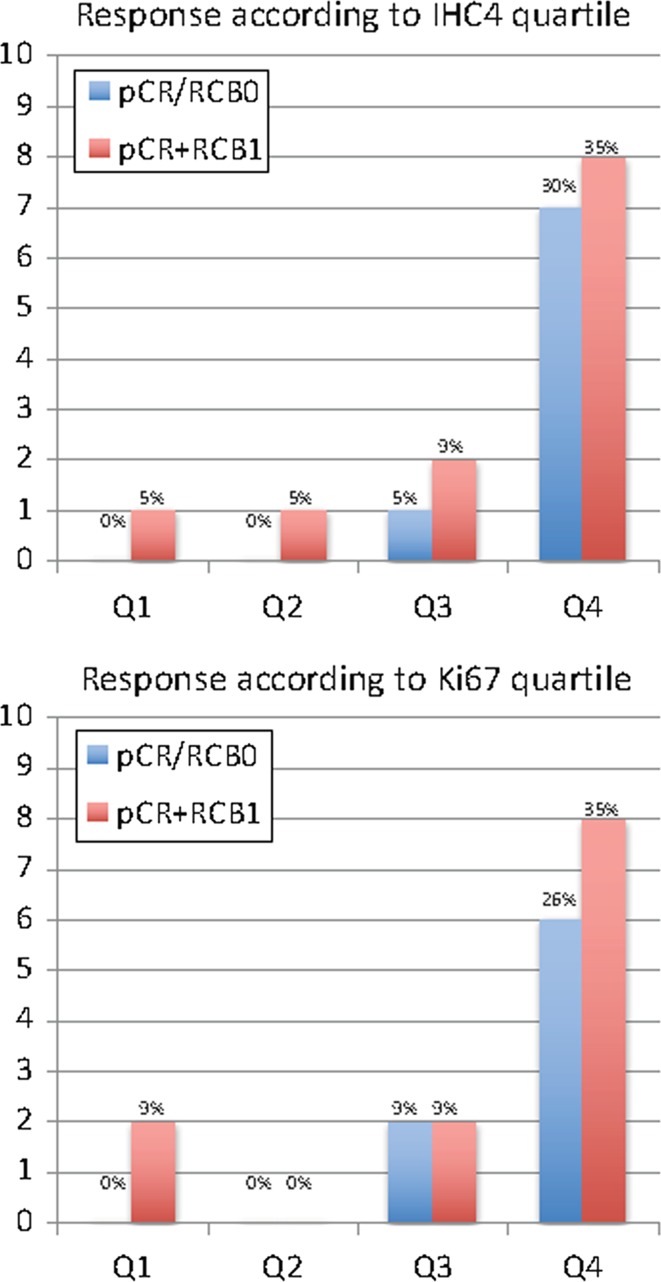
Pathological response according to IHC4 and Ki67 quartile for HER2−cases (*N* = 88)

Pathological response according to pre-treatment IHC4 score and Ki67 is shown for all cases and for HER2-negative cases in Figs. 1 and 2, respectively.

Eighteen (16%) of the 113 patients including 8 (9%) of the 88 HER2-negative cases achieved pCR (Figs. 1 and 2). Ki67 and IHC4 score were both positively associated with achievement of pCR (*P* < 10^−7^ and *P* < 10^−9^, respectively) and pCR+RCB1 (*P* < 10^−5^ and *P* < 10^−9^, respectively) following neo-adjuvant chemotherapy in all patients. In the highest IHC4 quartile, 52% achieved a pCR and 66% achieved either a pCR or minimal residual disease (RCB 1). In the highest Ki67 quartile, 38 and 45% achieved pCR and pCR+RCB1, respectively (Fig. 1). In the sub-group of ER+HER2− cases where IHC4 would be most applicable in clinical practice, 30% of the highest quartile achieved pCR and 35% pCR+RCB1. In the highest quartile for Ki67 in HER2- cases, 26 and 35% were achieved in pCR and pCR+RCB1, respectively (Fig. 2). There were no pCRs in the lowest quartile of IHC4 or Ki67 for all cases or HER2− cases.

Higher Ki67 score was associated with a numerically higher incidence of complete clinical response with the 75% of cases with a Ki67 in the highest quartile attaining a complete clinical response. The relationship with partial clinical response was less clear as was the relationship between IHC4 score and clinical response (Online Resource 3).

### Relationship between IHC4 and outcome

There was a weak but statistically significant correlation between pre-treatment IHC4 score and post-treatment RCB score [Pearson’s *R* = −0.27 (*P* = 0.04)].

The relationship between the pre-treatment IHC4 score and long-term outcome in this chemotherapy-treated cohort is shown in Online Resource 4. There was a trend towards shorter TTR being associated with higher IHC4 quartile although this was not significant(*P* = 0.24) and importantly IHC4 has only been validated as a predictor of long-term outcome in patients not treated with chemotherapy.

## Discussion

In this cohort of ER+ patients treated with neo-adjuvant chemotherapy, a high IHC4 score was strongly predictive of a pCR or near pCR (RCB1). Ki67 was an important component of this predictive ability, but IHC4 provided greater information. Whilst IHC4 has previously been shown to be prognostic, this study now demonstrates the relationship between IHC4 and response to chemotherapy. Both Ki67 and IHC4 were strongly predictive of pCR (*P* < 10^−7^ and *P* < 10^−9^, respectively).

A higher IHC4 score is associated with a poorer long-term outcome in the patients treated with adjuvant endocrine therapy alone, but in this chemotherapy-treated cohort baseline IHC4 did not show the same relationship. This indirectly suggests the hypothesis that chemotherapy benefit is focused to the group of patients with high IHC4 scores.

The relationship between other multigene signatures associated with prognosis in ER+ breast cancer and response to neo-adjuvant chemotherapy has also been examined by others. Oncotype DX has been tested in the neo-adjuvant setting and found to be associated with pCR (*P* = 0.005) [10]. Other signatures have also been associated with better response to neo-adjuvant chemotherapy although not necessarily survival benefit [15–18]. The EP signature was specifically tested in ER+, HER2− tumours where the benefits of chemotherapy are less certain. EP classification was associated with a pCR rate of 7% and in the low-risk group and 17% in the high-risk group (*P* = 0.001) with EP score significantly associated with a pCR [15]. The Prosigna risk of recurrence score (ROR) has also been shown to be correlated with response to neo-adjuvant chemotherapy in ER+ HER2− breast cancer. This includes a proliferation score, which was also strongly associated with response [18]. The findings in this study that a higher IHC4 score is associated with a higher rate of pCR are therefore consistent with other reports that patients at higher risk, as estimated from molecular profiles, respond better to chemotherapy.

Higher pre-treatment Ki67 was also associated with a greater likelihood of pCR but not all studies have shown Ki67 to be an independent predictor of pCR [19]. This may in part relate to analytical variability between labs assessing Ki67. Reports have examined the relationship between pre-treatment Ki67 in predicting benefit from adjuvant chemotherapy. Ki67 alone has not been shown to predict benefit of adjuvant cyclophosphamide/methotrexate/fluorouracil chemotherapy to adjuvant endocrine therapy in lymph node-negative patients [20]. Other studies have examined the potential of baseline Ki67 to predict benefit from specific regimes reporting a trend towards benefit from the addition of taxanes to anthracycline-based chemotherapy [21, 22].

A limitation to the assessment of chemotherapy benefit in ER+ disease in the neo-adjuvant setting is the heterogeneous outcome associated with residual disease, with pCR being a stronger predictor of long-term outcome in ER− or HER2+ disease [23]. In the current study, achievement of minimal residual disease (RCB class 1) was also assessed as this has been shown to be associated with excellent long-term outcome. However, further validation studies are required to define clinically useful cut-offs of risk for RCB in ER+ and ER− disease.

Ki67 scores in the highest quartile were associated with a 75% complete clinical response rate; however, there was no clear association between the IHC4 score in predicting clinical response to neo-adjuvant chemotherapy. Clinical response to neo-adjuvant chemotherapy is accepted to be a poor correlate of chemotherapy response, and this study was underpowered to address this question fully with complete information on clinical response only available in 85% of cases.

This study was limited by its relatively modest size and single institution, retrospective design. Thirty percent of cases assessed for RCB did not have pre-treatment samples available for analysis due to a significant proportion of cases being diagnosed at outside centres (S1). Nonetheless, RCB related to long-term outcome as expected and therefore this cohort may be fairly considered as representative of a contemporary ER+ neo-adjuvant population. The pathological work-up and reporting of residual disease was rigorous and allowed reconstruction of the residual tumour bed for assessment of RCB. The quantitative assessment of ER, PgR, and Ki67 were performed in a laboratory with extensive experience of conducting and assessing these results for standardized input for the IHC4 algorithm and the relationships of both Ki67+IHC4 were very strong. Efforts are ongoing to standardize IHC4 for wider adoption into clinical practice [24]. In conclusion, both pre-treatment Ki67 and IHC4 were positively and strongly associated with achievement of pCR with a greater association with IHC4 than pre-treatment Ki67 alone. These findings provide substantial support for IHC4 measured at baseline prior to neo-adjuvant chemotherapy providing predictive information on the responsiveness of tumours to chemotherapy.

## Electronic supplementary material

Below is the link to the electronic supplementary material.

Supplementary material 1 Patient cohort (TIFF 5 kb)

Supplementary material 2 Relationship between long-term outcome and RCB class. (TIFF 102 kb)

Supplementary material 3 Clinical response according to pre-treatment IHC4 and Ki67. *CR* complete response, *MRD* minimal residual disease, *PR* partial response, *NC*: no change. (TIFF 444 kb)

Supplementary material 4 Time to relapse (TTR) according to IHC4 quartile (TIFF 825 kb)

## References

[1] Peto R, Davies C, Godwin J (2012). Comparisons between different polychemotherapy regimens for early breast cancer: meta-analyses of long-term outcome among 100,000 women in 123 randomised trials. Lancet.

[2] Dowsett M, Goldhirsch A, Hayes DF (2007). International web-based consultation on priorities for translational breast cancer research. Breast Cancer Res.

[3] Cuzick J, Dowsett M, Pineda S (2011). Prognostic value of a combined estrogen receptor, progesterone receptor, Ki-67, and human epidermal growth factor receptor 2 immunohistochemical score and comparison with the Genomic Health recurrence score in early breast cancer. J Clin Oncol.

[4] Gene expression profiling and expanded immunohistochemistry tests for guiding adjuvant chemotherapy decisions in early breast cancer management: Mammaprint, Oncotype DX, IHC4 and Mammostrat. In NICE (ed). 2013

[5] Yeo B, Zabaglo L, Hills M (2015). Clinical utility of the IHC4+C score in oestrogen receptor-positive early breast cancer: a prospective decision impact study. Br J Cancer.

[6] Paik S, Tang G, Shak S (2006). Gene expression and benefit of chemotherapy in women with node-negative, estrogen receptor-positive breast cancer. J Clin Oncol.

[7] Ring AE, Smith IE, Ashley S (2004). Oestrogen receptor status, pathological complete response and prognosis in patients receiving neoadjuvant chemotherapy for early breast cancer. Br J Cancer.

[8] Colleoni M, Minchella I, Mazzarol G (2000). Response to primary chemotherapy in breast cancer patients with tumors not expressing estrogen and progesterone receptors. Ann Oncol.

[9] Kuerer HM, Newman LA, Smith TL (1999). Clinical course of breast cancer patients with complete pathologic primary tumor and axillary lymph node response to doxorubicin-based neoadjuvant chemotherapy. J Clin Oncol.

[10] Gianni L, Zambetti M, Clark K (2005). Gene expression profiles in paraffin-embedded core biopsy tissue predict response to chemotherapy in women with locally advanced breast cancer. J Clin Oncol.

[11] Symmans WF, Peintinger F, Hatzis C (2007). Measurement of residual breast cancer burden to predict survival after neoadjuvant chemotherapy. J Clin Oncol.

[12] Sheri A, Smith IE, Johnston SR (2015). Residual proliferative cancer burden to predict long-term outcome following neoadjuvant chemotherapy. Ann Oncol.

[13] Miller AB, Hoogstraten B, Staquet M, Winkler A (1981). Reporting results of cancer treatment. Cancer.

[14] Barton S, Zabaglo L, A’Hern R (2012). Assessment of the contribution of the IHC4+ C score to decision making in clinical practice in early breast cancer. Br J Cancer.

[15] Bertucci F, Finetti P, Viens P, Birnbaum D (2014). EndoPredict predicts for the response to neoadjuvant chemotherapy in ER-positive, HER2-negative breast cancer. Cancer Lett.

[16] Straver ME, Glas AM, Hannemann J (2010). The 70-gene signature as a response predictor for neoadjuvant chemotherapy in breast cancer. Breast Cancer Res Treat.

[17] Liedtke C, Hatzis C, Symmans WF (2009). Genomic grade index is associated with response to chemotherapy in patients with breast cancer. J Clin Oncol.

[18] Prat A, Galvan P, Jimenez B (2016). Prediction of response to neoadjuvant chemotherapy using core needle biopsy samples with the prosigna assay. Clin Cancer Res.

[19] Yerushalmi R, Woods R, Ravdin PM (2010). Ki67 in breast cancer: prognostic and predictive potential. Lancet Oncol.

[20] Viale G, Regan MM, Maiorano E (2008). Chemoendocrine compared with endocrine adjuvant therapies for node-negative breast cancer: predictive value of centrally reviewed expression of estrogen and progesterone receptors–International Breast Cancer Study Group. J Clin Oncol.

[21] Penault-Llorca F, Andre F, Sagan C (2009). Ki67 expression and docetaxel efficacy in patients with estrogen receptor-positive breast cancer. J Clin Oncol.

[22] Hugh J, Hanson J, Cheang MC (2009). Breast cancer subtypes and response to docetaxel in node-positive breast cancer: use of an immunohistochemical definition in the BCIRG 001 trial. J Clin Oncol.

[23] Cortazar P, Zhang L, Untch M (2014). Pathological complete response and long-term clinical benefit in breast cancer: the CTNeoBC pooled analysis. Lancet.

[24] Dodson A, Zabaglo L, Yeo B (2016). Risk of recurrence estimates with IHC4+ C are tolerant of variations in staining and scoring: an analytical validity study. J Clin Pathol.

