# Supplementary figures and images for: Relationship between IHC4 score and response to neo-adjuvant chemotherapy in estrogen receptor-positive breast cancer
Source: Breast Cancer Res Treat. 2017 Apr 26;164(2):395–400. doi: 10.1007/s10549-017-4266-9 (PMC5487724; doi:10.1007/s10549-017-4266-9)

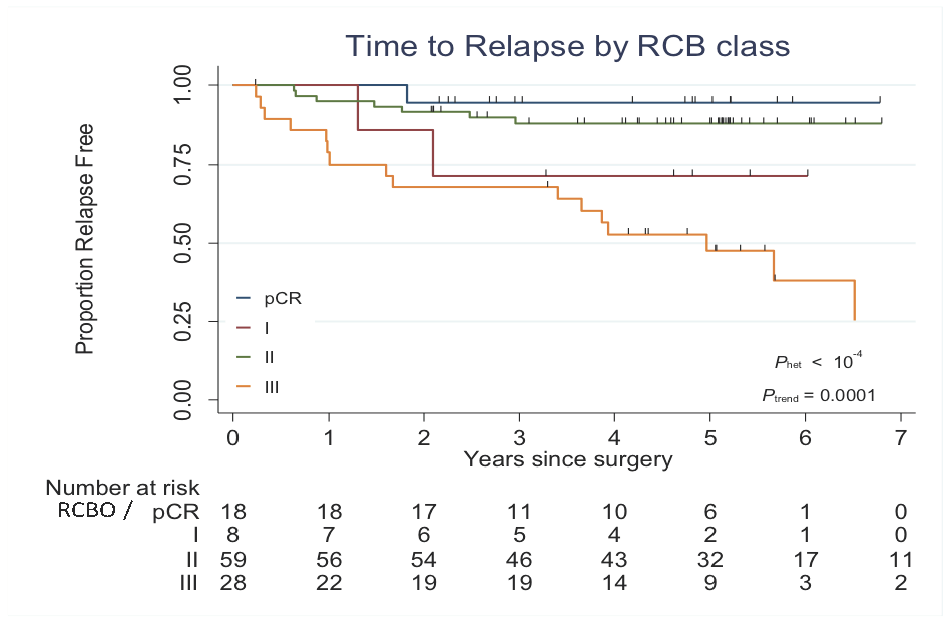

Supplement: Supplementary file 2 — Supplementary material 2 Relationship between long-term outcome and RCB class. (TIFF 102 kb) [file 10549_2017_4266_MOESM2_ESM.tiff]

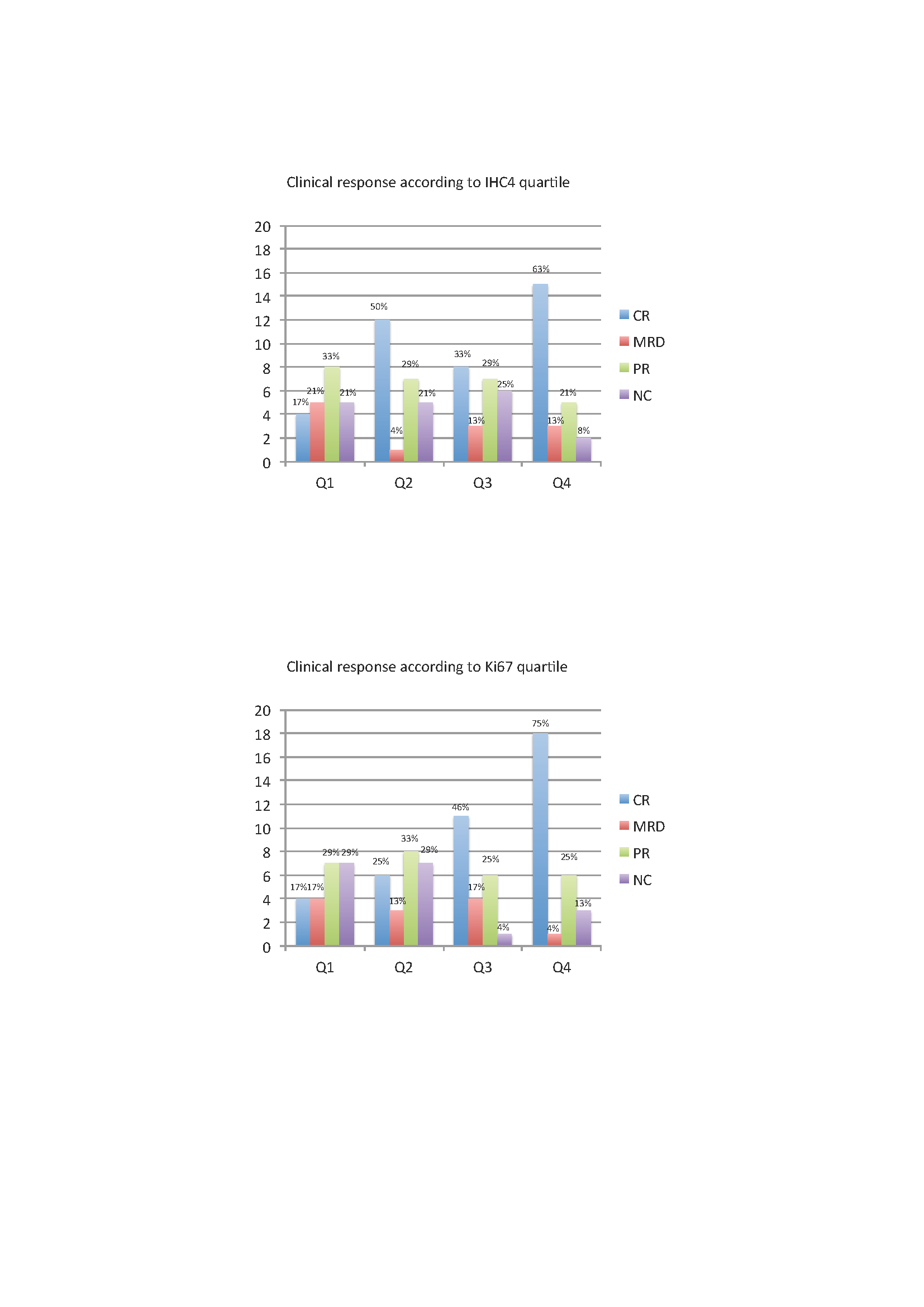

Supplement: Supplementary file 3 — Supplementary material 3 Clinical response according to pre-treatment IHC4 and Ki67. CR complete response, MRD minimal residual disease, PR partial response, NC: no change. (TIFF 444 kb) [file 10549_2017_4266_MOESM3_ESM.tiff]

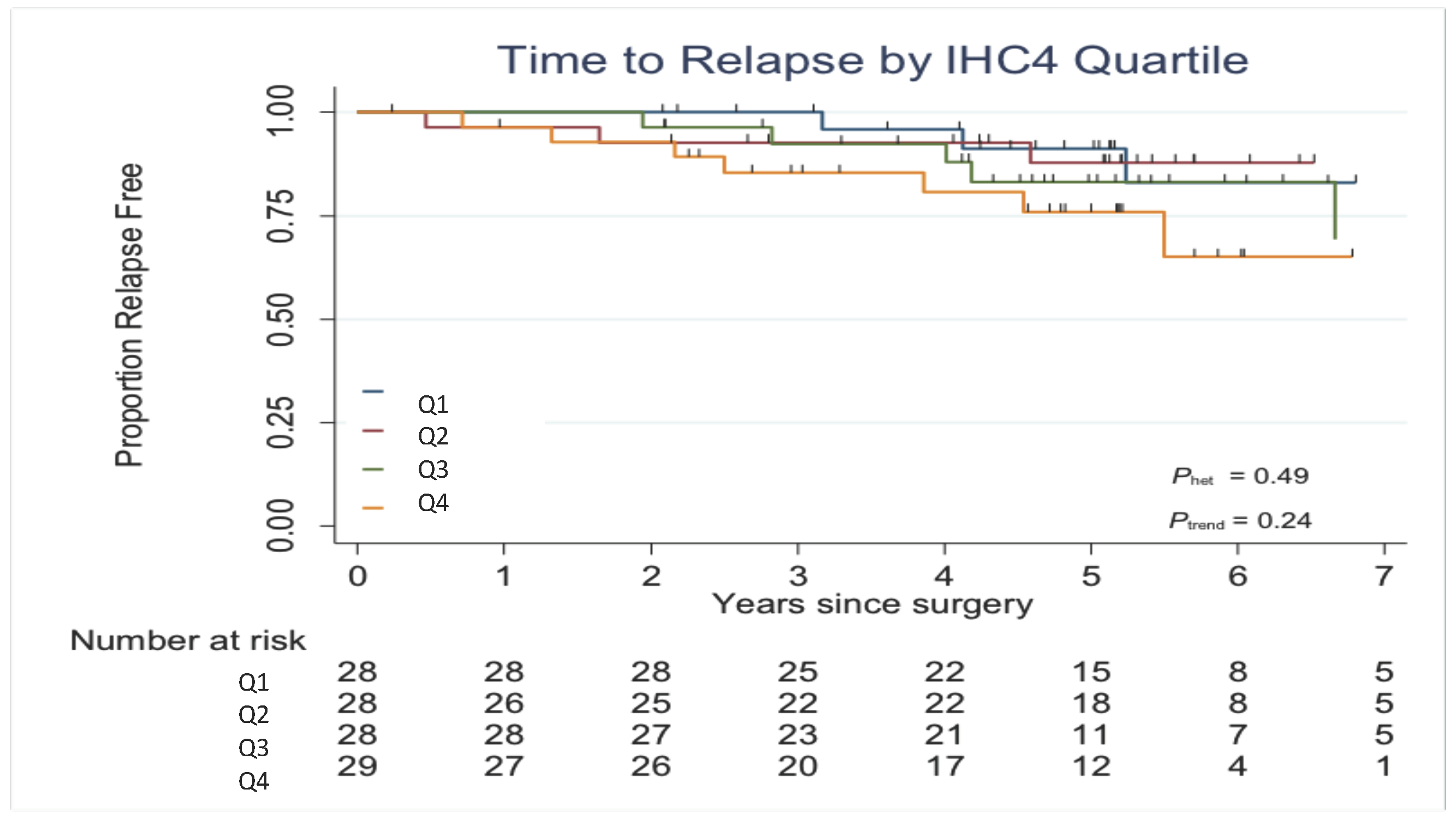

Supplement: Supplementary file 4 — Supplementary material 4 Time to relapse (TTR) according to IHC4 quartile (TIFF 825 kb) [file 10549_2017_4266_MOESM4_ESM.tiff]
